# Optimal timing of coronary angiograms for patients with chronic kidney disease: association between the duration of kidney dysfunction and SYNTAX scores

**DOI:** 10.1080/0886022X.2021.1880936

**Published:** 2021-02-04

**Authors:** Bei Song, Daopeng Dai, Shengjun Liu, Zhengbin Zhu, Fenghua Ding, Jinzhou Zhu, Ruiyan Zhang

**Affiliations:** Department of Vascular & Cardiology, Ruijin Hospital, Shanghai Jiao Tong University School of Medicine, Shanghai, P.R. China

**Keywords:** Chronic kidney disease, coronary artery disease, kidney dysfunction duration, SYNTAX scores

## Abstract

**Background:**

Chronic kidney disease (CKD) is associated with an increased risk of the progression of coronary artery disease (CAD). However, there are few data on the relationship between CAD severity and the duration of CKD. This study assessed the predictive value of the duration of kidney dysfunction in CKD patients with CAD severity.

**Methods:**

In 145 patients (63.4% male, *n* = 92; mean age, 68.8 ± 12.8 years) with CKD, severity of CAD was assessed by coronary angiography and quantified by SYNTAX scores, and duration of kidney dysfunction was either assessed by checking historical biochemical parameters of individuals or was based on enquiries.

**Results:**

Patients with high SYNTAX scores (≥ 22) had a greater prevalence of cardiovascular risk factors including age, gender, history of heart failure and smoking. In CKD patients, SYNTAX scores were positively correlated to duration of CKD and serum uric acid (UA), and negatively correlated to high-density lipoprotein-cholesterol (HDL-C) and ApoA1 levels. Univariate binary logistic regression and multivariate logistic analyses showed that SYNTAX scores correlated significantly with CKD duration, UA, and HDL-C. Receiver-operating characteristic analysis was used to explore a time point when coronary angiography application was economical and effective and yielded a Youden index of 6.5 years.

**Conclusions:**

Together, our results demonstrated that the duration of kidney dysfunction was an independent correlate of the severity of CAD in patients with CKD. Our findings suggest that coronary angiography should be considered for CKD patients with renal insufficiency having lasted for more than 6.5 years.

## Introduction

Chronic kidney disease (CKD) is reported to accelerate cardiovascular disease (CVD), and CVD is major cause of death in CKD patients [1–3]. In CKD, CVD manifests mainly as coronary artery disease (CAD) [4]. There is an increased prevalence of CAD as kidney function deteriorates, even at earlier stages of impaired kidney function [5–8].

CKD accelerates CAD *via* several mechanisms. Along with the classical risk factors of hypertension, dyslipidemia, smoking, diabetes mellitus, and aging [9–11], CKD-specific non-classical risk factors contribute to CAD. These include inflammation, volume overload, oxidative stress, the renin–angiotensin system, sympathetic nervous system, and uremic toxins [12]. In addition, CKD alters calcium and phosphorus homeostasis, resulting in hypercalcemia and vascular calcification, including of the coronary arteries [13].

However, it remains unclear how coronary plaques progress over time in CKD patients. The clinical diagnosis of CAD in CKD patients is challenging, as these patients do not demonstrate typical symptoms or electrocardiogram (ECG) changes. Additionally, CKD patients are hesitant to receive coronary angiographies because of potential adverse renal effects of contrast agents. As a result, CKD patients may miss the optimal time for interventional diagnosis and therapy of CAD, resulting in a higher cardiovascular morbidity and mortality.

To better understand how renal function influences coronary plaque progression, the present study investigated the relationship between clinical parameters and the severity of coronary lesions. Here, we introduced SYNTAX scores as a quantitative index for coronary artery disease. The SYNTAX score is a comprehensive angiographic scoring system that was initially designed to quantify CAD lesions based on the location, complexity, and functional impact [14]. Furthermore, the SYNTAX score is used to assist with patient selection for optimal revascularization strategies between PCI and CABG [15]. In this study, we assessed if the duration of kidney dysfunction predicts and correlates with the severity of coronary artery lesions. Additionally, we calculated the statistically recommended timing of coronary angiographies in CKD patients.

## Methods

### Patients

The study population comprised 145 patients with CKD (defined as glomerular filtration rate (GFR) < 60 mL/min/1.73 m^2^ for three months or more) admitted to the Rui Jin Hospital in Shanghai, China, between January 2013 and December 2018. GFR was estimated using the Chronic Kidney Disease Epidemiology Collaboration (CKD-EPI) formula [14]. Each patient underwent a coronary angiograph using the Judkins technique in multiple angulated views. Patients were excluded if there was a previous history of myocardial infarction, coronary artery bypass graft (CABG) surgery, and/or percutaneous coronary intervention (PCI). Patients were also excluded if they had diabetes mellitus, a malignant tumor, familial hypercholesterolemia, systemic disease (e.g. systemic lupus erythematosus), and/or LV systolic dysfunction defined as an LV ejection fraction ≤ 50%. The underlying renal diseases in the study cohort were hypertensive nephropathy (*n* = 53), chronic glomerulonephritis (*n* = 34), chronic interstitial nephritis (*n* = 11), polycystic kidney disease (*n* = 3), and other/unknown renal diseases (*n* = 44). The protocol in this study was approved by the institutional ethics committee of Ruijin Hospital (ID: 2020-152), Shanghai, China.

### Data collection

Body mass index (BMI) was calculated based on the height and weight of each patient. Hypertension was defined as either having a blood pressure ≥ 140/90 mmHg from at least two measurements, or the active use of an antihypertensive treatment. A history of smoking was defined as a patient reporting a history of active smoking within the last six months. Venous blood samples were collected from all patients in the morning after an overnight fast. Biochemical parameters were measured by standard laboratory procedures (Hitachi 7600 autoanalyzer, Roche Modular; Hitachi Ltd, Tokyo, Japan) and included the following: creatinine (CRE), urea nitrogen (BUN), uric acid (UA), albumin (ALB), white blood cells (WBCs), platelets (PLTs), hemoglobin (Hb), alanine aminotransferase (ALT), aspartate aminotransferase (AST), triglycerides (TGs), total cholesterol (TC), high-density lipoprotein-cholesterol (HDL-C), low-density lipoprotein-cholesterol (LDL-C), bilirubin, calcium, phosphate, C-reactive protein (CRP), fasting glucose, and B-type natriuretic peptide (BNP).

Two methods were used to assess the duration of kidney dysfunction in each patient. First, the duration of kidney dysfunction was assessed and calculated by checking the historical biochemical parameters of patients. Second, each patient was asked how many years earlier he/she had been diagnosed with kidney dysfunction.

### Coronary angiographies and SYNTAX scores

All patients underwent coronary angiographies through the radial artery using the Judkins technique. Each coronary artery was displayed on at least two different planes. All coronary angiograms were recorded on compact disks in DICOM format and were assessed independently by two experienced interventional cardiologists who were blinded to the study protocol and patient characteristics. In the case of disagreement, the opinion of a senior interventional cardiologist was sought, and the final decision was made by consensus. The SYNTAX score for each patient was calculated based on all coronary lesions with *a* ≥ 50% diameter of stenosis in vessels with a segment length >1.5 mm using the SYNTAX score algorithm available on the SYNTAX website (www.syntaxscore.com) [16,17].

### Statistical analysis

Data were analyzed using SPSS 13.0 (SPSS Inc., Chicago, IL, USA). Continuous variables are presented as the mean ± standard deviation (SD), whereas categorical variables are presented as percentages. The total group of patients was dichotomized based on SYNTAX scores (≥22 or <22; median as cutoff) for descriptive and analytical statistics. The missing data of each variable comprised less than 5% of all data, and listwise deletion was applied when data were analyzed. Student’s *t*-tests were used for the comparison of continuous variables while Chi-square tests were used for the comparison of categorical variables. Spearman and Pearson tests were used to evaluate correlations between SYNTAX scores and other variables. Multivariate logistic regression analysis was used for multivariate analysis of independent variables. Receiver-operating characteristic (ROC) analysis was used to determine the cutoff value of CKD duration in the prediction of high SYNTAX scores (≥ 22). A value of *p* < 0.05 was considered statistically significant.

## Results

### Clinical findings

Table 1 depicts the baseline characteristics of the 145 analyzed patients. There were significant differences in age, gender, heart failure and smoking between the high-SYNTAX-score (≥22) and low-SYNTAX-score (<22) groups, which reflected the traditional cardiovascular risk factors of CAD. More specifically, the CKD duration was significantly shorter in the low-SYNTAX-score group than in the high-SYNTAX-score group. Clinical and biochemical parameters of the study population are presented in Table 2. Compared to patients with low SYNTAX scores, patients with high SYNTAX scores had higher serum UA (*p* = 0.025) and lower HDL-C (*p* = 0.021) and Apo A1 (*p* = 0.018). In contrast, there were no significant differences in any other biochemical characteristics between the two groups. Of note, GFR was not a strong predictor of higher SYNTAX scores in the present study and conflicted with a previous study [18].

**Table 1.** Baseline characteristics of the study population.

Parameters	S*S* < 22 (*n* = 99)	S*S* ≥ 22 (*n* = 46)	*p* Value
SS	5.44 ± 6.76	34.28 ± 9.17	<0.001
Age (years)	67.3 ± 13.5	72.4 ± 10.39	0.014
BMI (kg/m^2^)	23.53 ± 3.12	24.19 ± 3.80	0.275
Gender (male%)	54(54.5%)	38(82.6%)	0.001
History of HBP (%)	73(73.7%)	37(80.4%)	0.380
History of HF (%)	3(3%)	8(17.4%)	0.002
History of alcohol consumption (%)	7(7.1%)	7(15.2%)	0.122
History of smoking (%)	16(16.2%)	18(39.1%)	0.002
Duration of CKD (years)	4(3–7)	12(9–15)	<0.001

Results are expressed as mean ± SD, median (quartile), or as number (frequency) for binary variables. SS: SYNTAX score; BMI: body mass index; HBP: high blood pressure; HF: heart failure; CKD: chronic kidney disease.

**Table 2.** Clinical and demographic characteristics of the study population.

Parameters	S*S* < 22 (*n* = 99)	S*S* ≥ 22 (*n* = 46)	*p* Value
WBCs (10^9^/L)	6.70 ± 1.90	7.22 ± 2.86	0.2020
HGB (g/L)	118 ± 20.8	111 ± 19.6	0.094
PLTs (10^9^/L)	165 ± 83.5	171 ± 65.5	0.680
ALT (IU/L)	22.4 ± 18.6	18.5 ± 11.0	0.193
AST (IU/L)	25.4 ± 16.0	45.5 ± 100.2	0.182
ALB (g/L)	35.6 ± 5.95	34.8 ± 4.69	0.455
BUN (mmol/L)	12.4 ± 6.78	14.3 ± 9.44	0.169
CRE (umol/L)	287.8 ± 287.8	298.2 ± 298.6	0.843
UA (umol/L)	437.6 ± 143.5	498.1 ± 162.4	0.025
Calcium (mmol/L)	2.21 ± 0.19	2.16 ± 0.22	0.316
Phosphate (mmol/L)	1.39 ± 0.47	1.35 ± 0.50	0.719
TGs (mmol/L)	1.79 ± 2.24	1.67 ± 0.88	0.732
TC (mmol/L)	4.00 ± 1.10	3.87 ± 1.13	0.543
HDL-C (mmol/L)	1.07 ± 0.27	0.96 ± 0.21	0.021
LDL-C (mmol/L)	2.27 ± 0.90	2.32 ± 0.98	0.774
Apo A1 (g/L)	1.16 ± 0.20	1.07 ± 0.19	0.018
Apo B (g/L)	0.78 ± 0.26	0.79 ± 0.29	0.811
Lp (a) (g/L)	0.34 ± 0.47	0.38 ± 0.34	0.662
FBG (mmol/L)	5.21 ± 1.57	5.19 ± 0.82	0.919
CK-MB (ng/mL)	4.89 ± 16.02	12.67 ± 57.57	0.373
cTnI (ng/mL)	2.41 ± 11.3	15.61 ± 88.7	0.320
CRP (mg/L)	16.0 ± 30.9	35.3 ± 78.1	0.139
BNP (pg/mL)	6762.1 ± 13121.6	6847.3 ± 10012.6	0.970
APTT (s)	31.0 ± 5.3	31.61 ± 6.3	0.578
PT (s)	13.3 ± 5.8	12.5 ± 2.7	0.413
FGB (mg/L)	4.38 ± 9.81	4.88 ± 3.68	0.762
DD (mg/L)	1.53 ± 5.25	1.31 ± 1.28	0.802
GFR (mL/min/1.73 m2)	29.84 ± 14.18	30.55 ± 13.74	0.778

Results are expressed as the mean ± SD. WBC: white blood cells; HGB: hemoglobin; PLTs: platelets; ALT: alanine aminotransferase; AST: aspartate aminotransferase; ALB: albumin (ALB); BUN: blood urea nitrogen; CRE: creatinine; UA: uric acid; TGs: triglycerides; TC: total cholesterol; HDL-C: high-density lipoprotein-cholesterol; LDL-C: low-density lipoprotein-cholesterol; Apo: apolipoprotein; Lp: lipoprotein; FBG: fasting blood glucose; cTnI: cardiac troponin I; CRP: C-reactive protein; BNP: B-type natriuretic peptide; FGB: fibrin degradation products; DD: D-dimer; GFR: glomerular filtration rate.

### CKD duration is significantly associated with SYNTAX score in CKD patients

**Figure 1.**
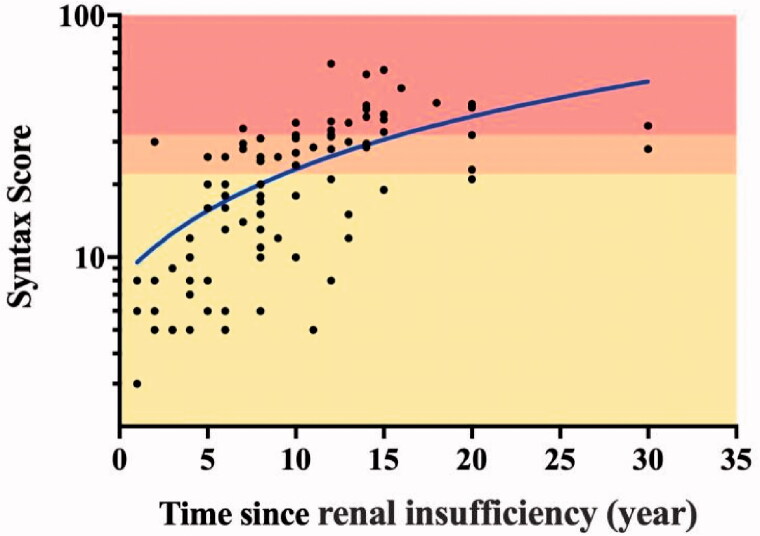
Correlation between the duration of kidney dysfunction and CAD defined by SYNTAX scores.

**Figure 2.**
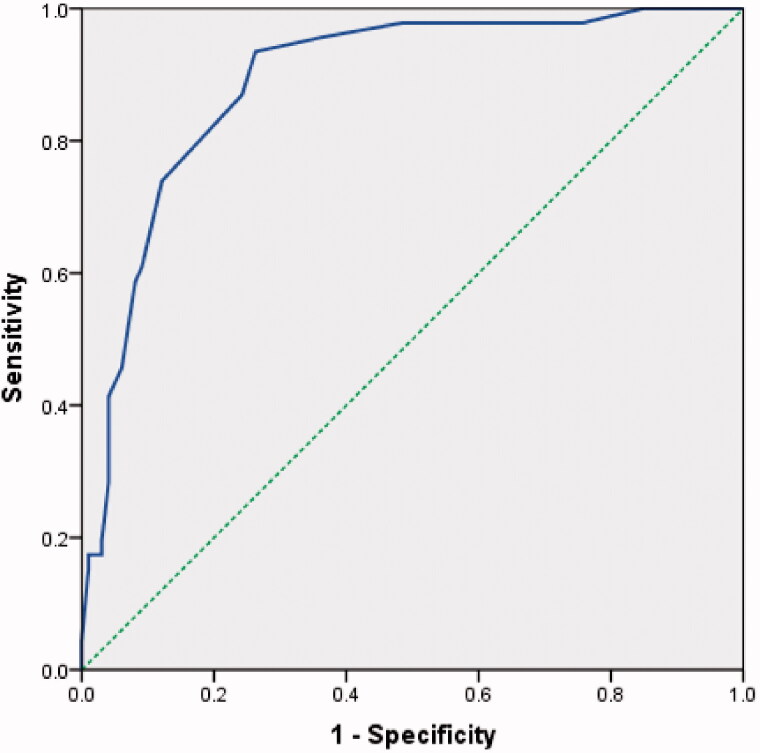
ROC curves for determining the cutoff level of CKD duration for a SYNTAX score ≥ 22. The area under the ROC curve was 0.889 (*p* < 0.001). The Youden index was 6.5 (years since renal insufficiency), with a specificity of 0.737 and a sensitivity of 0.935.

The results of univariate binary logistic regression and multivariate logistic analyses to predict the severity of CAD in CKD patients are listed in Table 3. SYNTAX scores correlated significantly with CKD duration (*β* = 0.378, *p* < 0.001), UA (*β* = 0.004, *p* = 0.023), and HDL-C (*β* = −2.724, *p* = 0.023). In unadjusted analysis, CKD duration and SYNTAX scores were positively correlated (Figure 1). According to ROC analysis, 6.5 years (since renal insufficiency), with a specificity of 0.737 and sensitivity of 0.935, was determined to be the best time point to predict a SYNTAX score of 22, at which time coronary angiographies would have been considered for the patients with symptoms or a clinical presentation concerning CAD (Figure 2).

**Table 3.** Binary logistic regression analyses for SYNTAX scores ≥ 22.

Parameters	β	*p*	OR (95% CI)
Univariate			
Duration of CKD (years)	0.346	<0.001	1.413 (1.260–1.584)
Age (years)	0.035	0.028	1.035 (1.004–1.068)
Gender (male%)	1.376	0.002	3.958 (1.677–9.344)
History of HF (%)	1.908	0.007	6.737 (1.696–26.753)
History of smoking (%)	1.204	0.003	3.335 (1.501–7.408)
UA (μmol/L)	0.003	0.031	1.003 (1.000–1.005)
HDL-C (mmol/L)	−1.835	0.024	0.160 (0.032–0.790)
ApoA (g/L)	−2.221	0.021	0.108 (0.017–0.712)
Multivariate model 1 Nagelkerke *R*^2^ 0.570			
Duration of CKD (years)	0.378	<0.001	1.459 (1.281–1.662)
UA (μmol/L)	0.004	0.023	1.004 (1.001–1.008)
HDL-C (mmol/L)	−2.724	0.023	0.066 (0.006–0.691)
Constant	−3.252	0.040	0.39
Multivariate model 2 Nagelkerke *R*^2^ 0.521			
Duration of CKD (years)	0.347	<0.001	1.415 (1.242–1.611)
UA (μmol/L)	0.004	0.023	1.004 (1.001–1.008)
HDL-C (mmol/L)	−2.465	0.038	0.085 (0.008–0.857)
Constant	−3.278	0.040	0.038

UA: uric acid; HDL-C: high-density lipoprotein-cholesterol.

Model 1 included age, gender, history of HF, history of smoking, UA, HDL-C, ApoA, and duration of CKD (years).

Model 2 included age, gender, history of HBP, history of HF, history of smoking, Cre, BUN, UA, TC, LDL-C, HDL-C, ApoA, CRP, and duration of CKD (years).

## Discussion

Studies show that coronary severity is associated with CKD. To the best of our knowledge, a relationship between the severity of CAD and the duration of CKD has not been reported previously. In this retrospective study, we introduced SYNTAX scores as a quantitative index for coronary artery disease. We showed that SYNTAX scores were significantly associated with CKD duration, UA, and HDL-C. Our results revealed that long-term CKD was a strong predictor of higher SYNTAX scores. ROC was used to explore a time point and yielded a Youden index of 6.5 years. Thus, for patients with a history of renal insufficiency of 6.5 years or longer and clinical symptoms related to CAD, coronary angiography should be considered.

CKD is closely related with CVD [19,20]. It is noteworthy that even at earlier stages of CVD, CKD is associated with a higher incidence of cardiovascular morbidity and mortality [21,22]. CAD severity is also an independent risk factor for decline in kidney function [23]. The high prevalence of CAD cannot be completely explained by the presence of traditional risk factors such as age, hypertension, dyslipidemia, diabetes mellitus, smoking, and family history [3]. This suggests there are yet to be identified risk factors for CAD, some of which may be unique to CKD. Novel risk factors, such as serum calcitonin [24], low-grade inflammation [25], multifunctional molecules (e.g. TWEAK and MCP-1) [26,27], and endothelial dysfunction [28], were reported to be highly associated with advanced CAD in patients with kidney disease. Putative nontraditional risk factors also include activation of the renin–angiotensin system and sympathetic nerve activity, as well as reduced bioavailability of nitric oxide, platelet dysfunction, vitamin D deficiency, and hyperphosphatemia [22]. Our previous studies have shown that the uremic toxin PCS not only promoted atherosclerotic lesion formation and induced plaque instability, but also promoted the injury of myocardial cells and was associated with the progression of diastolic dysfunction [29,30]. In the present study, we showed that long-term accumulation of CKD was correlated with the aggravation of atherosclerosis.

Given the high prevalence of CAD in patients with CKD, it is paradoxical that the diagnosis and treatment of CAD in these individuals is low. The universal determination of CAD has only a limited application in patients with CKD. Chest discomfort and ECG abnormalities may not reflect CAD, and CKD patients may have chronic baseline increases in specific cardiac biomarker levels [31]. Thus, more accurate identification methods and risk-stratification strategies are needed [32]. Furthermore, due to concerns for contrast-induced nephropathy (CIN), aggressive treatments for CAD involving PCI have been performed less frequently [33] and thus might contribute to the high mortality rate of CKD patients. In the present study, we found that 6.5 years since the start of renal insufficiency yielded good accuracy and specificity for the determination of a SYNTAX score reaching 22, as determined by ROC analysis. Thus, when deciding to cath a patient with symptoms or a clinical presentation concerning for CAD, a patient with CKD for greater than 6.5 years should be taken into account and may lead the patient toward a more invasive strategy. Of note, measures to avoid CIN should be taken. To avoid the interference of traditional risk factors that promote atherosclerosis in CKD patients, patients with diabetes mellitus, familial hypercholesterolemia, and autoimmune diseases were excluded in the present study. Statistical analysis was then performed after adjustment for age, gender, and smoking. We found that the duration of kidney dysfunction was still correlated with atherosclerosis severity after adding patients with diabetes mellitus (data not shown). This result might be due to the strong disadvantageous effects of CKD on atherosclerosis, as a previous study has shown that the coronary risk conferred by CKD even surpasses that of diabetes [34].

Our present study also showed reduced HDL-C and elevated serum UA levels to be important risk factors for the aggravation of atherosclerosis. HDL-C plays a protective role against developing CAD in several ways. ApoA1 is a primary protein constituent of HDL in plasma. Studies showed that HDL reduced oxidative damage and inflammation, enhanced endothelial function, removed excess cholesterol from macrophages, and exerted protective effects on angiogenesis and glucose homeostasis [35,36]. UA, a component of metabolic syndrome and a risk factor for CAD, is also a major contributor to the development and progression of CKD. Many studies confirmed an association of elevated serum UA levels with the incidence of CAD, but the causality in the UA-CAD relationship remains unproven [37]. The role of UA in CAD will remain a fascinating research field in the future.

Our present study had several limitations. First, the etiology of CKD in each patient was unclear, and few diagnoses were confirmed by renal biopsy. Second, the duration of kidney dysfunction was largely obtained from enquiries, which may have caused data inaccuracy. Third, this was a single-center retrospective case study based on a relatively small number of patients in China. Despite these limitations, our study demonstrated, for the first time, that the duration of kidney dysfunction was associated with atherosclerosis severity in patients with CKD, as determined by coronary angiographies and SYNTAX scores. Taken together, our findings may help to better understand the relationship between CKD and CAD and provide clinical insight for the treatment of CAD in CKD patients. Further research is needed to identify whether angiograms in all patients with CKD of 6.5 years is beneficial in a prospective fashion.

## References

[1] Go AS, Chertow GM, Fan D, et al. Chronic kidney disease and the risks of death, cardiovascular events, and hospitalization. N Engl J Med. 2004;351(13):1296–1305. 15385656 10.1056/NEJMoa041031

[2] Parikh NI, Hwang SJ, Larson MG, et al. Chronic kidney disease as a predictor of cardiovascular disease (from the Framingham Heart Study). Am J Cardiol. 2008;102(1):47–53. 18572034 10.1016/j.amjcard.2008.02.095 PMC2517213

[3] Cheung AK, Sarnak MJ, Yan G, et al. Atherosclerotic cardiovascular disease risks in chronic hemodialysis patients. Kidney Int. 2000;58(1):353–362. 10886582 10.1046/j.1523-1755.2000.00173.x

[4] Tangri N, Komenda PV, Rigatto C. Chronic kidney disease and heart disease: after 179 years, do we yet understand the link? Kidney Int. 2015;88(1):11–13. 26126089 10.1038/ki.2015.112

[5] Bae EH, Lim SY, Cho KH, et al. GFR and cardiovascular outcomes after acute myocardial infarction: results from the Korea Acute Myocardial Infarction Registry. Am J Kidney Dis. 2012;59(6):795–802. 22445708 10.1053/j.ajkd.2012.01.016

[6] Nakano T, Ninomiya T, Sumiyoshi S, et al. Association of kidney function with coronary atherosclerosis and calcification in autopsy samples from Japanese elders: the Hisayama study. Am J Kidney Dis. 2010;55(1):21–30. 19765871 10.1053/j.ajkd.2009.06.034

[7] Betriu A, Martinez-Alonso M, Arcidiacono MV, et al. Prevalence of subclinical atheromatosis and associated risk factors in chronic kidney disease: the NEFRONA study. Nephrol Dial Transplant. 2014;29(7):1415–1422. 24586070 10.1093/ndt/gfu038

[8] Adeseun GA, Xie D, Wang X, et al. Carotid plaque, carotid intima-media thickness, and coronary calcification equally discriminate prevalent cardiovascular disease in kidney disease. Am J Nephrol. 2012;36(4):342–347. 23107930 10.1159/000342794 PMC3538165

[9] Baigent C, Burbury K, Wheeler D. Premature cardiovascular disease in chronic renal failure. Lancet. 2000;356(9224):147–152. 10963260 10.1016/S0140-6736(00)02456-9

[10] Vaziri ND. Dyslipidemia of chronic renal failure: the nature, mechanisms, and potential consequences. Am J Physiol Renal Physiol. 2006;290(2):F262–72. 16403839 10.1152/ajprenal.00099.2005

[11] Wilson PWF, D’Agostino RB, Levy D, et al. Prediction of coronary heart disease using risk factor categories. Circulation. 1998;97(18):1837–1847. 9603539 10.1161/01.cir.97.18.1837

[12] Sarnak MJ, Levey AS, Schoolwerth AC, et al. Kidney disease as a risk factor for development of cardiovascular disease: a statement from the American Heart Association Councils on Kidney in Cardiovascular Disease, High Blood Pressure Research, Clinical Cardiology, and Epidemiology and Prevention. Circulation. 2003;108(17):2154–2169. 14581387 10.1161/01.CIR.0000095676.90936.80

[13] Hage FG, Venkataraman R, Zoghbi GJ, et al. The scope of coronary heart disease in patients with chronic kidney disease. J Am Coll Cardiol. 2009;53(23):2129–2140. 19497438 10.1016/j.jacc.2009.02.047

[14] Sianos G, Morel MA, Kappetein AP, et al. The SYNTAX Score: an angiographic tool grading the complexity of coronary artery disease. EuroIntervention. 2005;1:219–227. 19758907

[15] Serruys PW, Morice MC, Kappetein AP, et al. Percutaneous coronary intervention versus coronary-artery bypass grafting for severe coronary artery disease. N Engl J Med. 2009;360(10):961–972. 19228612 10.1056/NEJMoa0804626

[16] Levey AS, Stevens LA, Schmid CH, et al. A new equation to estimate glomerular filtration rate. Ann Intern Med. 2009;150(9):604–612. 19414839 10.7326/0003-4819-150-9-200905050-00006 PMC2763564

[17] Serruys PW, Onuma Y, Garg S, et al. Assessment of the SYNTAX score in the Syntax study. EuroIntervention. 2009;5(1):50–56. 19577983 10.4244/eijv5i1a9

[18] Coskun U, Orta Kilickesmez K, Abaci O, et al. The relationship between chronic kidney disease and SYNTAX score. Angiology. 2011;62(6):504–508. 21422054 10.1177/0003319711398864

[19] Sarnak MJ, Amann K, Bangalore S, et al. Chronic kidney disease and coronary artery disease: JACC state-of-the-art review. J Am Coll Cardiol. 2019;74(14):1823–1838. 31582143 10.1016/j.jacc.2019.08.1017

[20] Matsushita K, van der Velde M, Astor BC, et al. Association of estimated glomerular filtration rate and albuminuria with all-cause and cardiovascular mortality in general population cohorts: a collaborative meta-analysis. Lancet. 2010;375(9731):2073–2081. 20483451 10.1016/S0140-6736(10)60674-5 PMC3993088

[21] Manjunath G, Tighiouart H, Ibrahim H, et al. Level of kidney function as a risk factor for atherosclerotic cardiovascular outcomes in the community. J Am Coll Cardiol. 2003;41(1):47–55. 12570944 10.1016/s0735-1097(02)02663-3

[22] Gansevoort RT, Correa-Rotter R, Hemmelgarn BR, et al. Chronic kidney disease and cardiovascular risk: epidemiology, mechanisms, and prevention. Lancet. 2013;382(9889):339–352. 23727170 10.1016/S0140-6736(13)60595-4

[23] Turak O, Afsar B, Siriopol D, et al. Severity of coronary artery disease is an independent risk factor for decline in kidney function. Eur J Intern Med. 2016;33:93–97. 27406080 10.1016/j.ejim.2016.06.031

[24] Kanbay M, Wolf M, Selcoki Y, et al. Association of serum calcitonin with coronary artery disease in individuals with and without chronic kidney disease. Int Urol Nephrol. 2012;44(4):1169–1175. 22130958 10.1007/s11255-011-0076-x

[25] Kanbay M, Ikizek M, Solak Y, et al. Uric acid and pentraxin-3 levels are independently associated with coronary artery disease risk in patients with stage 2 and 3 kidney disease. Am J Nephrol. 2011;33(4):325–331. 21389698 10.1159/000324916 PMC3064941

[26] Azak A, Akdoğan MF, Denizli N, et al. Soluble TWEAK levels are independently associated with coronary artery disease severity in patients with stage 2-3 kidney disease. Int Urol Nephrol. 2014;46(2):411–415. 24043442 10.1007/s11255-013-0562-4

[27] Akdoğan MF, Azak A, Denizli N, et al. MCP-1 and soluble TWEAK levels are independently associated with coronary artery disease severity in patients with chronic kidney disease. Renal Failure. 2015;37(8):1297–1302. 26382008 10.3109/0886022X.2015.1065428

[28] Stenvinkel P, Carrero JJ, Axelsson J, et al. Emerging biomarkers for evaluating cardiovascular risk in the chronic kidney disease patient: how do new pieces fit into the uremic puzzle? Clin J Am Soc Nephrol. 2008;3(2):505–521. 18184879 10.2215/CJN.03670807 PMC6631093

[29] Han H, Chen Y, Zhu Z, et al. p-Cresyl sulfate promotes the formation of atherosclerotic lesions and induces plaque instability by targeting vascular smooth muscle cells. Front Med. 2016;10(3):320–329. 27527366 10.1007/s11684-016-0463-x

[30] Han H, Zhu J, Zhu Z, et al. p-Cresyl sulfate aggravates cardiac dysfunction associated with chronic kidney disease by enhancing apoptosis of cardiomyocytes. J Am Heart Assoc. 2015;4(6):e001852. 26066032 10.1161/JAHA.115.001852 PMC4599533

[31] Khan NA, Hemmelgarn BR, Tonelli M, et al. Prognostic value of troponin T and I among asymptomatic patients with end-stage renal disease: a meta-analysis. Circulation. 2005;112(20):3088–3096. 16286604 10.1161/CIRCULATIONAHA.105.560128

[32] Shroff GR, Chang TI. Risk stratification and treatment of coronary disease in chronic kidney disease and end-stage kidney disease. Semin Nephrol. 2018;38(6):582–599. 30413253 10.1016/j.semnephrol.2018.08.004

[33] Lau JK, Anastasius MO, Hyun KK, et al. Evidence-based care in a population with chronic kidney disease and acute coronary syndrome. Findings from the Australian Cooperative National Registry of Acute Coronary Care, Guideline Adherence and Clinical Events (CONCORDANCE). Am Heart J. 2015;170(3):566–572.e1. 26385041 10.1016/j.ahj.2015.06.025

[34] Tonelli M, Muntner P, Lloyd A, et al. Risk of coronary events in people with chronic kidney disease compared with those with diabetes: a population-level cohort study. Lancet. 2012;380(9844):807–814. 22717317 10.1016/S0140-6736(12)60572-8

[35] Nicholls SJ, Nelson AJ. HDL and cardiovascular disease. Pathology. 2019;51(2):142–147. 30612759 10.1016/j.pathol.2018.10.017

[36] Rosenson RS, Brewer HB, Jr, Ansell BJ, et al. Dysfunctional HDL and atherosclerotic cardiovascular disease. Nat Rev Cardiol. 2016;13(1):48–60. 26323267 10.1038/nrcardio.2015.124 PMC6245940

[37] Ndrepepa G. Uric acid and cardiovascular disease. Clin Chim Acta. 2018;484:150–163. 29803897 10.1016/j.cca.2018.05.046

